# Prognostic Impact of Residual Renal Congestion at Discharge After Transcatheter Aortic Valve Implantation

**DOI:** 10.1016/j.cjco.2026.02.016

**Published:** 2026-02-27

**Authors:** Yutaro Sato, Akihiko Sato, Kazuya Sakamoto, Yuuki Muto, Yu Sato, Tetsuro Yokokawa, Takeshi Shimizu, Tomofumi Misaka, Masayoshi Oikawa, Atsushi Kobayashi, Akiomi Yoshihisa, Yasuchika Takeishi

**Affiliations:** a Department of Cardiovascular Medicine, Fukushima Medical University, Fukushima, Japan; b Department of Clinical Laboratory Sciences, Fukushima Medical University School of Health Sciences, Fukushima, Japan

**Keywords:** aortic stenosis, transcatheter aortic valve implantation, intrarenal venous flow, renal congestion, cardiorenal syndrome, heart failure

## Abstract

**Background:**

Residual pulmonary congestion after transcatheter aortic valve implantation (TAVI) has been linked to adverse outcomes. However, whether renal congestion, assessed by intrarenal venous flow (IRVF), is associated with outcomes remains unclear.

**Methods:**

This study enrolled 164 patients with aortic stenosis who underwent TAVI, and assessed IRVF before TAVI and at discharge (48-72 hours before discharge). The IRVF patterns were categorized as continuous (non-congestive) or discontinuous (congestive). The primary endpoint was a composite of all-cause death or heart failure rehospitalization.

**Results:**

Before TAVI, 126 patients (76.8%) had a continuous pattern, and 38 (23.2%) had a discontinuous pattern; at discharge, the numbers were 134 (81.7%) and 30 (18.3%), respectively. Over a median follow-up of 698 days, the primary endpoint occurred in 37 patients (22.6%). A discontinuous pattern at discharge was associated with a higher incidence of events than a continuous pattern (log-rank *P* = 0.005), whereas the pre-TAVI IRVF pattern did not stratify risk. In an analysis of changes in IRVF patterns (continuous–continuous, continuous–discontinuous, discontinuous–continuous, and discontinuous–discontinuous), event rates were 20 of 114 (17.5%), 5 of 12 (41.7%), 4 of 20 (20.0%), and 8 of 18 (44.4%), respectively, and the risk was higher in patients discharged with a discontinuous pattern—continuous–discontinuous (hazard ratio [HR], 3.26, 95% confidence interval [CI] 1.19-8.92; *P* = 0.022) and discontinuous–discontinuous (HR, 2.34, 95% CI 1.08-5.37; *P* = 0.045)—compared with those in the continuous–continuous group.

**Conclusions:**

A discontinuous IRVF pattern at discharge, indicating residual renal congestion after TAVI, was associated with an adverse prognosis.

## Résumé

**Contexte:**

La congestion pulmonaire résiduelle après une implantation valvulaire aortique par cathéter (IVAC) est associée à un pronostic défavorable. On ne sait toutefois pas avec certitude si c’est aussi le cas de la congestion rénale évaluée en fonction du flux veineux intrarénal (FVIR).

**Méthodologie:**

Cette étude a été menée chez 164 patients présentant une sténose aortique ayant subi une IVAC, chez qui le FVIR a été évalué avant l’intervention et au moment du congé de l’hôpital (de 48 à 72 heures avant le congé de l’hôpital). Les profils de FVIR ont été classés selon qu’ils étaient continus (absence de congestion) ou discontinus (congestion). Le critère d’évaluation principal était composite et combinait la mortalité toutes causes confondues et la réhospitalisation pour insuffisance cardiaque (IC).

**Résultats:**

Avant l’IVAC, 126 patients (76,8 %) présentaient un profil continu et 38 patients (23,2 %), un profil discontinu; au moment du congé de l’hôpital, ils étaient respectivement 134 (81,7 %) et 30 (18,3 %). Pendant un suivi médian de 698 jours, le critère d’évaluation principal a été observé chez 37 patients (22,6 %). Un profil discontinu au moment de quitter l’hôpital a été associé à une incidence plus élevée des événements, comparativement à un profil continu (valeur *p* selon le test logarithmique par rangs = 0,005), alors que le profil de FVIR avant l’IVAC n’a pas permis de stratifier ce risque. Dans une analyse des variations du profil de FVIR (continu-continu, continu-discontinu, discontinu-continu et discontinu-discontinu), les taux d’événements ont été de 20/114 (17,5 %), 5/12 (41,7 %), 4/20 (20,0 %) et 8/18 (44,4 %), respectivement, et le risque a été plus élevé chez les patients ayant un profil discontinu au moment du congé de l’hôpital : continu-discontinu (rapport des risques instantanés [RRI] : 3,26; intervalle de confiance [IC] à 95 % : 1,19 à 8,92; *p* = 0,022) et discontinu-discontinu (RRI : 2,34; IC à 95 % : 1,08 à 5,37; *p* = 0,045), comparativement au risque dans le groupe correspondant au profil continu-continu.

**Conclusions:**

Un profil de FVIR discontinu au moment du congé de l’hôpital, qui indique la présence d’une congestion rénale résiduelle après une IVAC, est associé à un pronostic défavorable.

Aortic stenosis (AS) is one of the most prevalent valvular heart diseases in older adults, and it is associated with adverse outcomes, including heart failure (HF) and sudden cardiac death.^1^ Transcatheter aortic valve implantation (TAVI) is a less-invasive alternative to surgical aortic valve replacement, with safety and efficacy demonstrated across high-, intermediate-, and low-risk populations.^2^, ^3^, ^4^ In patients undergoing TAVI, congestion at admission is associated with multiorgan dysfunction and a higher risk of all-cause death and HF rehospitalization,^5^ and residual pulmonary congestion at discharge has been reported to predict adverse outcomes.^6^ After TAVI, pulmonary congestion often improves relatively early, with relief of left-sided outflow obstruction, whereas systemic and renal venous congestion may persist. Persistent right-sided hemodynamic load related to atrial fibrillation (AF), secondary mitral regurgitation (MR), or tricuspid regurgitation may therefore lead to a dissociation between pulmonary and renal congestion. Despite these clinical and pathophysiological considerations, no standardized method for assessing systemic and renal venous congestion after TAVI has been established.

In HF, reduced cardiac output and elevated right-sided pressures lead to prerenal hypoperfusion and renal venous congestion, both of which are pathophysiological hallmarks of cardiorenal syndrome.^7^ Renal congestion has been reported to be associated with impaired renal function and adverse outcomes, underscoring the need for timely assessment and effective decongestion.^8^ Accordingly, renal decongestion is a key therapeutic target in HF management.^7^^,^^8^

Intrarenal Doppler ultrasonography (IRD) provides a practical, noninvasive method to assess renal congestion by measuring intrarenal venous flow (IRVF)—a discontinuous IRVF pattern indicates renal congestion, reflecting elevated right-sided pressures and reduced renal venous compliance.^9^, ^10^, ^11^ In HF, a discontinuous IRVF pattern is associated with adverse outcomes, including all-cause death and HF rehospitalization.^9^ In patients with severe AS, TAVI may improve hemodynamics and cardiorenal syndrome–related abnormalities.^12^ To the best of our knowledge, IRVF patterns have not been serially evaluated before and after TAVI in relation to long-term outcomes. Therefore, the aims of the present study were to determine whether renal congestion after TAVI, defined by IRVF patterns, is associated with clinical outcomes, and to evaluate whether changes in IRVF patterns from before to after TAVI are associated with clinical outcomes.

## Methods

### Study population and protocol

**Figure 1:**
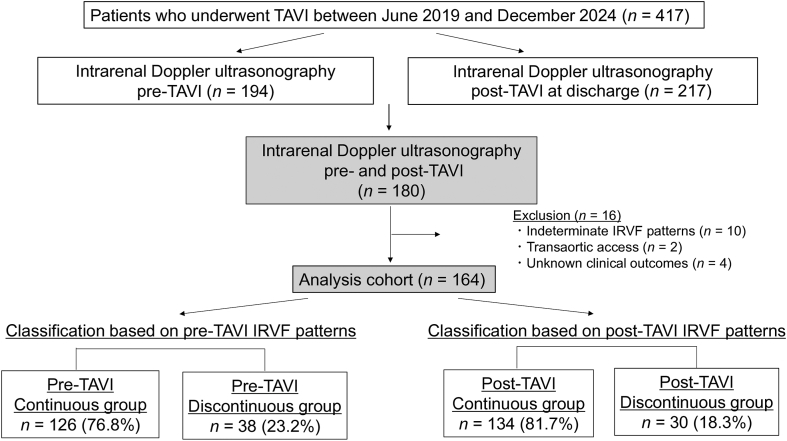
Flowchart of patient selection. IRVF, intrarenal venous flow; TAVI, transcatheter aortic valve implantation

**Figure 2:**
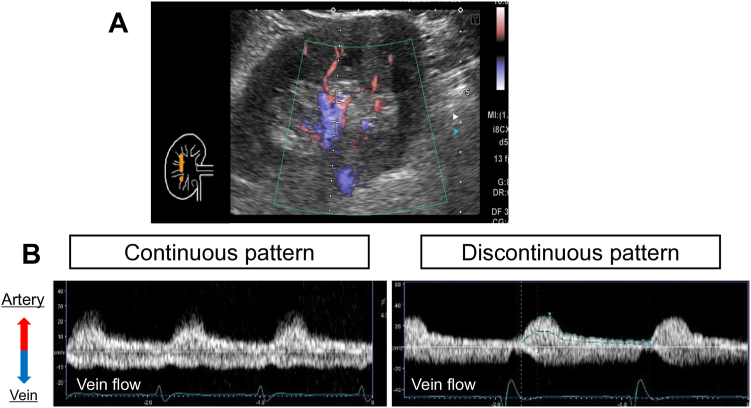
Intrarenal venous flow (IRVF) assessment with intrarenal Doppler ultrasonography. (**A**) Color Doppler imaging of IRVF. (**B**) Representative Doppler waveforms of IRVF, showing (**left**) a continuous pattern and (**right**) a discontinuous pattern.

The patient selection flowchart is presented in Figure 1. This prospective, observational study included 417 consecutive patients with severe AS who underwent TAVI at our institution between June 2019 and December 2024. IRD was performed pre-TAVI in 194 of these patients and post-TAVI in 217. Among these, 180 underwent IRD both pre- and post-TAVI. Patients who had indeterminate IRVF patterns (n = 10), underwent TAVI via transaortic access (n = 2), or had unknown clinical outcomes (n = 4) were excluded. Therefore, the final analysis cohort comprised 164 patients. IRVF patterns were categorized as continuous (normal, non-congestive) or discontinuous (congestive; Fig. 2).

First, we compared baseline clinical characteristics, laboratory data, echocardiographic parameters, and TAVI procedural characteristics between patients with continuous or discontinuous IRVF patterns at discharge (hereafter referred to as the “continuous group” and “discontinuous group,” respectively). Second, patients were followed until April 2025 for the composite endpoint of all-cause death or HF rehospitalization. HF rehospitalization was defined, consistent with Valve Academic Research Consortium-3 (VARC-3)^13^, as an unplanned hospitalization lasting at least 24 hours, primarily due to new or worsening HF that required escalation of HF therapy. Escalation of therapy included use of intravenous diuretics, intravenous vasodilators or inotropes, oxygen therapy, or noninvasive positive pressure ventilation. Planned admissions were excluded. Procedure- or valve-related rehospitalizations also were excluded unless the admission primarily reflected worsening HF requiring escalation of HF therapy. Time to the first event was analyzed, and patients without an event were censored at their last contact. We obtained vital status (including date of death) and HF rehospitalizations from institutional medical records; when records were insufficient, we confirmed vital status and HF rehospitalization events by telephone with the attending physicians at referring hospitals. Clinical events were adjudicated by physicians who were blinded to IRVF findings.

The study adhered to the Declaration of Helsinki and the Strengthening the Reporting of Observational Studies in Epidemiology (STROBE) statement. The protocol was approved by the Research Ethics Committee of Fukushima Medical University (approval No. 823, REC 2024-161). Written informed consent was obtained from all participants.

### IRVF assessment with IRD

IRVF was evaluated using an Aplio i800 ultrasound system (Canon Medical Systems, Tochigi, Japan) with a wideband convex i8CX1 probe (center frequency 4.0 MHz; range 1.8-6.4 MHz). Two experienced sonographers, blinded to clinical data and outcomes, performed all examinations, which were conducted pre-TAVI (within 30 days before TAVI), post-TAVI (within 48-72 hours before index discharge), or at both time points. Index discharge was defined as the first hospital discharge following the index TAVI procedure. Acquisition settings were standardized according to a predefined protocol. All patients fasted for ≥ 12 hours and were scanned in the left lateral decubitus position. The segmental renal vein was selected as the sampling site to maximize feasibility and reproducibility in our elderly TAVI cohort, because it was consistently visualized with an adequate Doppler angle and was assessed within the same acoustic window as the intrarenal arteries. The segmental renal vein of the right kidney was examined to avoid left-sided venous compression by the aorta or superior mesenteric artery and inflow from the left gonadal vein. Segmental vessels were identified by color Doppler (velocity scale, approximately 10-20 cm/s), and the pulsed-wave Doppler sample volume was positioned accordingly. The transducer was placed on the lateral abdominal wall; patients raised both arms over their chest to optimize the acoustic window. Measurements were obtained during breath-holding with an insonation angle of < 60°, while recording simultaneous waveforms of the segmental artery (upward) and vein (downward; Fig. 2A).

IRVF patterns were classified as continuous or discontinuous. Representative images of both patterns are shown in Figure 2B. The discontinuous pattern was defined as transient cessation of intrarenal venous flow during the cardiac cycle and was prespecified as indicative of renal congestion. In sinus rhythm, the most stable waveform from 5 consecutive cycles was analyzed; in AF, the index beat (the beat following 2 cycles of equal duration) was used.

### TAVI procedure and post-TAVI management

Severe AS was defined as an aortic valve area of < 1.0 cm^2^ with either a mean transvalvular gradient of > 40 mm Hg or a peak aortic jet velocity of > 4.0 m/s on transthoracic echocardiography.^14^ Procedural decisions—including access (transfemoral as the first choice) and valve type and size—were made by a multidisciplinary heart team, based on contrast-enhanced computed tomography findings. The devices used included balloon-expandable valves (Edwards SAPIEN 3 and/or SAPIEN 3 Ultra Resilia, Edwards Lifesciences, Irvine, CA) and self-expanding valves (Evolut R and/or PRO and/or PRO+ and/or FX, Medtronic, Minneapolis, MN). Procedures were performed under general anesthesia in a hybrid catheterization laboratory. Postprocedural antithrombotic therapy (single or dual antiplatelet therapy, or oral anticoagulation) was prescribed as clinically indicated. Patients were initially monitored in the cardiac care unit and then transferred to the general ward for guideline-directed rehabilitation and medical therapy.^14^

Technical success and device success were defined according to the VARC-3 criteria.^13^ Paravalvular leak (PVL) was graded according to VARC-3, with PVL ≥ moderate considered clinically significant.^13^

### Comorbidities and clinical definitions

Comorbidities were abstracted from medical records. Diagnoses were made by the treating physicians based on contemporary Japanese guideline criteria and medication use, when applicable. Hypertension was defined as the use of antihypertensive medication, systolic blood pressure of ≥ 140 mm Hg, and/or diastolic blood pressure of ≥ 90 mm Hg. Diabetes mellitus was defined as the use of insulin or oral antidiabetic agents, fasting plasma glucose of ≥ 126 mg/dL, and/or HbA1c of ≥ 6.5%. Dyslipidemia was defined as a low-density lipoprotein (LDL) cholesterol level ≥ 140 mg/dL or the use of lipid-lowering therapy. Chronic kidney disease (CKD) was defined as estimated glomerular filtration rate of < 60 mL/min per 1.73 m^2^. AF was diagnosed based on in-hospital electrocardiogram findings and/or prior medical records. Peripheral artery disease was defined as an ankle–brachial index of < 0.90 or ≥ 1.40. Prior stroke required a documented clinical diagnosis with imaging confirmation. Current smoking was defined as smoking at enrollment or within 6 months before hospitalization.

### Statistical analysis

Categorical variables are presented as numbers (percentages) and were compared using the χ^2^test or Fisher’s exact test. The normality of continuous variables was assessed with the Shapiro–Wilk test. Normally distributed variables are reported as mean ± standard deviation and were compared using the Student *t* test (2 groups) or 1-way analysis of variance (≥ 3 groups). Variance homogeneity was assessed with Levene’s test; when the variances were equal, Tukey’s post hoc tests were used, and when they were not equal, the Games–Howell test was used. Non-normally distributed variables are presented as median (interquartile range) and were compared using the Mann–Whitney *U* test (2 groups) or the Kruskal–Wallis test (≥ 3 groups).

Time-to-event outcomes were analyzed with Kaplan–Meier estimates and compared using the log-rank test. To adjust for potential confounding, inverse probability of treatment weighting (IPTW) using propensity scores was performed to evaluate the association between the IRVF pattern at discharge (post-TAVI; discontinuous vs continuous) and outcomes. Propensity scores for a discontinuous post-TAVI IRVF pattern were estimated using logistic regression, including age, sex, AF, clinical frailty scale (CFS), and hypertension, as well as B-type natriuretic peptide (BNP) level, MR ≥ moderate, and inferior vena cava (IVC) diameter at discharge. The association between the post-TAVI IRVF pattern and outcomes was evaluated using an IPTW-adjusted Cox proportional hazards model with robust (sandwich) standard errors. Covariate balance after weighting was assessed using standardized mean differences. As a sensitivity analysis, we performed a parsimonious multivariable Cox proportional hazards model adjusted for age, sex, AF, CFS score, and discharge BNP level. Prespecified subgroup analyses were performed. In subgroup analyses, categorical covariates were analyzed as present vs absent. Continuous covariates were dichotomized using established, clinically meaningful cutoffs whenever available. Because no established cutoff exists in this setting, BNP level was dichotomized at the cohort median for an interpretable, exploratory presentation. Subgroups were prespecified a priori based on established prognostic factors for aortic stenosis.^1^^,^^14^^,^^15^ Within each subgroup, hazard ratios (HRs) with 95% confidence intervals (CIs) were estimated, and interaction *P* values were derived from models including the IRVF pattern, the subgroup factor, and their multiplicative interaction term. In addition, a Fine–Gray competing-risk model was used for HF rehospitalization, treating all-cause death as a competing event, adjusted for age and sex. Subdistribution HRs with 95% CIs were estimated. For comparison, cause-specific Cox proportional hazards models were fitted separately for HF rehospitalization and all-cause death, adjusted for age and sex. Cause-specific HRs with 95% CIs were estimated. The proportional hazards assumption was assessed using Schoenfeld residuals. A 2-sided *P* < 0.05 was considered statistically significant. Missing data were handled using a complete-case approach for the propensity-score model in the IPTW analysis. Patients with missing values in covariates required for the propensity-score model were excluded. Echocardiographic variables with missing data were summarized descriptively in Table 1 and Supplemental Tables S1-S2. Incremental prognostic value was assessed by comparing a baseline model with and without the IRVF pattern using the C-index, likelihood ratio testing, integrated discrimination improvement, continuous net reclassification improvement, and decision-curve analysis. These statistical analyses were performed using SPSS (version 29.0, IBM, Armonk, NY). IPTW, competing-risk analyses (Fine–Gray and cause-specific Cox models) were performed using EZR (version 1.68, Saitama Medical Centre, Jichi Medical University, Saitama, Japan).

**Table 1:** Baseline patient characteristics stratified by post-transcatheter aortic valve implantation (TAVI) intrarenal venous flow (IRVF) pattern (n = 164)

Characteristic	Total (*n* = 164)	Continuous group (*n* = 134)	Discontinuous group (*n* = 30)	*P*
**Demographic data**				
Age, Y	83.4 ± 4.7	83.1 ± 4.7	84.7 ± 4.4	0.090
Female sex	106 (64.6)	85 (63.4)	21 (70.0)	0.496
Body mass index, kg/m^2^	22.7 (20.6–25.4)	22.9 (20.6–25.6)	21.7 (20.4–24.9)	0.087
NYHA class III or IV at discharge	3 (1.8)	2 (1.5)	1 (3.3)	0.457
STS score, %	4.8 (3.4–6.9)	4.7 (3.3–6.9)	6.1 (3.8–7.9)	0.039
Clinical frailty scale ≥ 4	56 (34.1)	40 (29.9)	16 (53.3)	0.019
**Past medical history**				
Hypertension	126 (76.8)	108 (80.6)	18 (60.0)	0.016
Diabetes mellitus	42 (25.6)	34 (25.4)	8 (26.7)	0.883
Dyslipidemia	71 (43.3)	57 (42.5)	14 (46.7)	0.680
Smoking	47 (28.7)	40 (29.9)	7 (23.3)	0.475
Peripheral artery disease	17 (10.4)	13 (9.7)	4 (13.3)	0.518
Chronic kidney disease	110 (67.1)	87 (64.9)	23 (76.7)	0.216
Previous stroke	8 (4.9)	8 (6.0)	0 (0.0)	0.353
AF	31 (18.9)	19 (14.2)	12 (40.0)	0.001
COPD	38 (23.2)	31 (23.1)	7 (23.3)	0.981
OMI	7 (4.3)	4 (3.0)	3 (10.0)	0.116
Previous PCI	20 (12.2)	15 (11.2)	5 (16.7)	0.372
Previous PMI	7 (4.3)	6 (4.5)	1 (3.3)	0.779
**Medication (post-TAVI)**				
Diuretics	68 (41.5)	49 (36.6)	19 (63.3)	0.007
ACE inhibitors or ARBs	81 (49.4)	69 (51.5)	12 (40.0)	0.255
Beta-blockers	44 (26.8)	33 (24.6)	11 (36.7)	0.179
Calcium channel blockers	90 (54.9)	80 (59.7)	10 (33.3)	0.009
**Laboratory data (post-TAVI)**				
BNP, pg/mL	115.3 (63.5–243.0)	100.5 (56.7–202.3)	252.9 (138.2–496.2)	< 0.001
eGFR, mL/min per 1.73 m^2^	55.0 (44.5–65.0)	55.0 (43.0–65.0)	51.0 (37.0–67.0)	0.261
Hemoglobin, g/dL	11.0 ± 1.5	11.1 ± 1.5	10.7 ± 1.4	0.155
Albumin, g/dL	3.4 (3.1–3.8)	3.4 (3.1–3.8)	3.4 (3.0–3.6)	0.476
Sodium, mEq/L	138.0 (136.0–140.0)	137.0 (135.0–139.0)	137.0 (136.0–139.0)	0.466
**Echocardiographic data (post-TAVI)**				
LVEF, %	61.0 (53.0–65.0)	61.0 (54.3–65.0)	63.0 (46.3–66.5)	0.141
E/e′	15.9 (11.8–19.7)	15.1 (11.5–19.1)	18.2 (16.1–22.3)	0.004
LAD, mm	44.0 (40.0–49.8)	43.0 (39.0–47.8)	47.0 (42.5–51.5)	< 0.001
Aortic valve area, cm^2^	1.72 (1.45–2.02)	1.79 (1.47–2.02)	1.51 (1.38–2.02)	0.083
AV peak velocity, m/s	2.0 (1.8–2.3)	2.0 (1.8–2.3)	1.8 (1.7–2.3)	0.148
AV mean pressure gradient, mm Hg	8.0 (6.0–11.0)	8.0 (6.0–11.0)	7.5 (6.0–11.0)	0.538
MR ≥ moderate	6 (3.7)	2 (1.5)	4 (13.3)	0.011
TRPG, mm Hg (n = 151)	23.0 (19.0–27.0)	22.0 (19.0–25.0)	27.0 (22.0–33.0)	< 0.001
TAPSE, mm (n = 160)	18.3 ± 4.2	18.5 ± 4.1	16.9 ± 4.1	0.051
IVC diameter, mm (n = 162)	14.6 ± 3.6	14.2 ± 3.1	16.7 ± 4.5	< 0.001
**Procedural characteristics**				
Procedural time, min	79.0 (64.3–96.8)	77.5 (64.0–95.0)	78.0 (57.3–99.0)	0.622
Length of hospital stay, d	12.0 (10.0–18.0)	12.0 (10.0–16.8)	14.5 (11.0–20.5)	0.031
Contrast, mL	67.0 (55.0–83.5)	67.5 (55.0–81.8)	66.5 (55.0–85.8)	0.846
Balloon-expandable valve	103 (62.8)	89 (66.4)	14 (46.7)	0.059
Self-expanding valve	61 (37.2)	45 (33.6)	16 (53.3)	0.059
**Procedural complications**				
Technical success	159 (97.0)	129 (96.3)	30 (100)	0.586
Device success	146 (89.0)	120 (89.6)	26 (86.7)	0.746
Myocardial infarction	1 (0.6)	1 (0.7)	0 (0.0)	0.421
Procedural stroke	3 (1.8)	3 (2.2)	0 (0.0)	0.776
Pacemaker implantation	7 (4.3)	6 (4.5)	1 (3.3)	0.825
PVL ≥ moderate	9 (5.5)	5 (3.7)	4 (13.3)	0.142

Values are mean ± standard deviation, n (%), or median (interquartile range), unless otherwise indicated.

ACE, angiotensin-converting enzyme; AF, atrial fibrillation; ARB, angiotensin II receptor blocker; AV, aortic valve; BNP, B-type natriuretic peptide; COPD, chronic obstructive pulmonary disease; E/e′, ratio of early mitral inflow velocity to mitral annular early diastolic velocity; eGFR, estimated glomerular filtration rate; IVC, inferior vena cava; LAD, left atrial diameter; LVEF, left ventricular ejection fraction; MR, mitral regurgitation; NYHA, New York Heart Association; OMI, old myocardial infarction; PCI, percutaneous coronary intervention; PMI, pacemaker implantation; PVL, paravalvular leak; STS, Society of Thoracic Surgeons; TAPSE, tricuspid annular plane systolic excursion; TRPG, tricuspid regurgitation pressure gradient.

**Table 2:** Cox proportional hazards and subgroup analyses for the composite outcome of all-cause death or heart failure rehospitalization (37 events/164 patients). Hazard ratios (HRs) compare the discontinuous vs continuous groups defined by the post-transcatheter aortic valve implantation intrarenal venous flow (TAVI IRVF) pattern at discharge

Variable	Subgroup	*N*	HR	95% CI	*P*	Interaction *P*
Age	≥ 80.0	135	2.227	1.093–4.540	0.027	0.301
	< 80.0	29	6.292	0.394–100.587	0.193	
Sex	Male	58	2.812	0.937–8.443	0.065	0.944
	Female	106	2.647	1.076–6.515	0.034	
BMI	≥ 20.0	134	4.086	1.860–8.978	< 0.001	0.077
	< 20.0	30	0.492	0.062–3.926	0.492	
STS score	≥ 8.0	26	1.460	0.346–6.158	0.606	0.592
	< 8.0	138	2.621	1.176–5.842	0.018	
CFS	≥ 4.0	56	1.848	0.775–4.408	0.166	0.610
	< 4.0	108	2.637	0.808–8.604	0.108	
CKD	(+)	110	1.997	0.977–4.083	0.068	0.462
	(−)	54	4.102	0.357–47.062	0.257	
AF	(+)	31	1.319	0.379–4.584	0.663	0.240
	(−)	133	3.117	1.368–7.100	0.007	
BNP	≥ 115.3	82	1.601	0.739–3.470	0.233	0.319
	< 115.3	82	3.867	0.790–18.927	0.095	
Albumin	≥ 3.5	78	4.856	1.359–17.353	0.015	0.290
	< 3.5	86	1.878	0.825–4.275	0.134	
LVEF	≥ 50.0	143	1.486	0.600–3.678	0.392	0.071
	< 50.0	21	10.992	1.337–92.535	0.027	
PVL ≥ moderate	(+)	9	1.349	0.219–8.316	0.747	0.688
	(−)	155	2.418	1.136–5.151	0.022	

AF, atrial fibrillation; BMI, body mass index; BNP, B-type natriuretic peptide; CFS, clinical frailty scale; CI, confidence interval; CKD, chronic kidney disease; LVEF, left ventricular ejection fraction; PVL, paravalvular leak; STS, Society of Thoracic Surgeons.

## Results

### IRVF Patterns

Patients were grouped according to their pre- and post-TAVI IRVF patterns. Pre-TAVI, 126 of 164 patients (76.8%) had a continuous pattern, and 38 (23.2%) had a discontinuous pattern; post-TAVI, these numbers were 134 (81.7%) and 30 (18.3%), respectively (Fig. 1).

### Baseline characteristics stratified by post-TAVI IRVF pattern

Baseline demographics, laboratory values, echocardiographic indices, and procedural characteristics stratified by post-TAVI IRVF pattern are presented in Table 1. For comparison, those stratified by pre-TAVI IRVF pattern are shown in Supplemental Table S1. Stratified by post-TAVI IRVF pattern, the continuous group comprised 134 patients, whereas the discontinuous group, which was interpreted as having residual renal congestion, comprised 30 patients. As shown in Table 1, age, sex, body mass index, and New York Heart Association (NYHA) functional class at discharge were similar between the groups. The Society of Thoracic Surgeons (STS) scores and the proportion of patients with a CFS score ≥ 4 were higher in the discontinuous group. Hypertension was more prevalent in the continuous group, whereas AF was more prevalent in the discontinuous group. The prevalence of diabetes mellitus, CKD, and other comorbidities was comparable between the groups. Diuretics were used more frequently in the discontinuous group, whereas calcium-channel blockers were used more frequently in the continuous group. BNP levels were higher in the discontinuous group, whereas estimated glomerular filtration rate (eGFR) was similar between the groups. At baseline (pre-TAVI), left ventricular ejection fraction (LVEF) and indices of AS severity were comparable (Supplemental Table S1). At discharge, echocardiography (Table 1) showed similar LVEF between groups; however, the discontinuous group had a higher E/e′ ratio, a larger left atrial diameter, a higher prevalence of ≥ moderate MR, a higher tricuspid regurgitation pressure gradient, and a larger IVC diameter. Hospital stay was longer in the discontinuous group, whereas contrast volume and technical/device success rates, as well as rates of permanent pacemaker implantation and ≥ moderate PVL, were similar between the groups.

### Clinical events during follow-up

**Figure 3:**
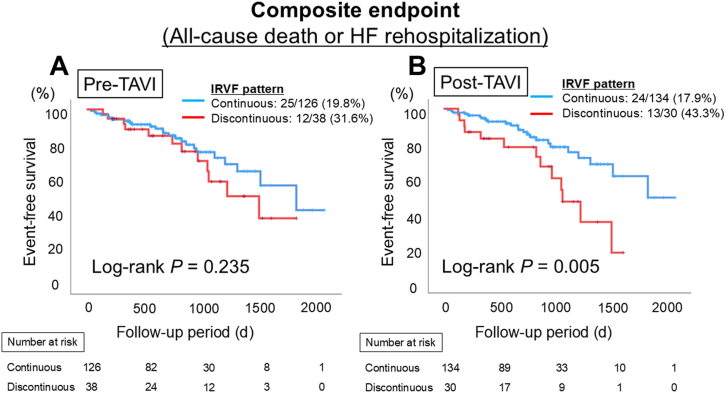
Composite endpoint by intrarenal venous flow (IRVF) pattern pre- and post-transcatheter aortic valve implantation (TAVI). Kaplan–Meier curves for event-free survival regarding the composite endpoint of all-cause death or heart failure (HF) rehospitalization, stratified by IRVF pattern. (**A**) Pre-TAVI, no significant difference was observed between the continuous and discontinuous groups (log-rank *P* = 0.235). (**B**) Post-TAVI, the discontinuous group had significantly lower event-free survival than the continuous group (log-rank *P* = 0.005). The numbers at risk are shown below each panel.

The median follow-up was 698 days (range, 38-2067 days). The primary composite endpoint (all-cause death or HF rehospitalization) occurred in 37 patients (22.6%). Kaplan–Meier analysis showed no significant difference in event-free survival between groups stratified by the pre-TAVI IRVF pattern (Fig. 3A; log-rank *P* = 0.235), indicating no association between pre-procedural renal congestion and the primary endpoint. In contrast, when stratified by the post-TAVI IRVF pattern, event-free survival was lower in the discontinuous group (Fig. 3B; log-rank *P* = 0.005), suggesting an association between residual renal congestion at discharge and an increased risk of the primary endpoint. Consistently, in univariable Cox analysis, a post-TAVI discontinuous IRVF pattern was associated with a higher risk of the primary endpoint (hazard ratio [HR], 2.58, 95% confidence interval [CI] 1.30-5.11; *P* = 0.007). In the IPTW-adjusted Cox model, a post-TAVI discontinuous IRVF pattern remained significantly associated with a higher risk of the primary endpoint (HR 2.52, 95% CI 1.19-5.34, *P* = 0.016). Covariate balance after weighting was acceptable, with all absolute standardized mean differences < 0.10 (Supplemental Fig. S1). Propensity-score distributions showed reasonable overlap, and the distribution of IPTW weights is presented in Supplemental Figure S2. Truncation of IPTW weights at the 1st and 99th percentiles resulted in findings consistent with the primary analysis (HR 2.43, 95% CI 1.16-5.11, *P* = 0.019). A parsimonious multivariable Cox model showed a directionally consistent association, although it did not reach statistical significance (HR 1.90, 95% CI 0.89-4.06, *P* = 0.099). Prespecified subgroup analyses stratified by age, sex, body mass index, STS score, CFS score, CKD, AF, BNP level, albumin, LVEF, and PVL ≥ moderate (Table 2) showed consistent associations, with no significant interactions (all *P*-values for interaction > 0.05). Additionally, exploratory subgroup analyses of other variables likewise showed no significant interactions (Supplemental Table S3).

HF rehospitalization occurred in 7 of 30 patients (23.3%) in the discontinuous group, and 7 of 134 (5.2%) in the continuous group, whereas all-cause death occurred in 7 of 30 (23.3%) and 20 of 134 (14.9%), respectively. In the Fine–Gray competing-risk model, treating all-cause death as a competing event and adjusting for age and sex, the post-TAVI discontinuous IRVF pattern was associated with a higher cumulative incidence of HF rehospitalization than the continuous pattern (HR, 3.23, 95% CI 1.01-10.35; *P* = 0.019; Supplemental Table S4). Similar findings were observed in the cause-specific Cox model for HF rehospitalization adjusting for age and sex (HR, 4.31, 95% CI 1.37-13.52; *P* = 0.012). By contrast, the post-TAVI discontinuous IRVF pattern was not significantly associated with all-cause death in the cause-specific Cox model (HR, 1.07, 95% CI 0.42-2.70; *P* = 0.892; Supplemental Table S4).

### Changes in IRVF pattern from pre- to post-TAVI

**Figure 4:**
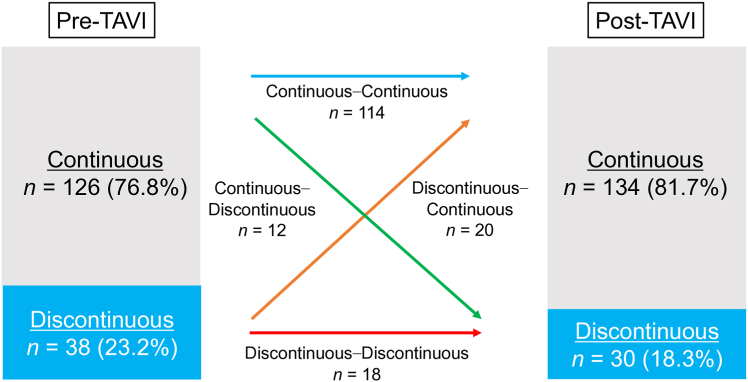
Changes in intrarenal venous flow pattern from pre- to post-transcatheter aortic valve implantation (TAVI). Patients were categorized into 4 groups: continuous–continuous (*n* = 114; 69.5%), discontinuous–continuous (*n* = 20; 12.2%), continuous–discontinuous (*n* = 12; 7.3%), and discontinuous–discontinuous (*n* = 18; 11.0%).

**Figure 5:**
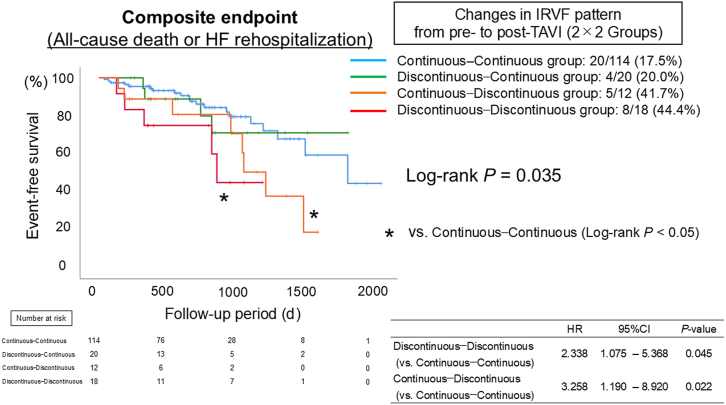
Composite endpoint by change in intrarenal venous flow (IRVF) pattern from pre- to post-transcatheter valve implantation (TAVI). Kaplan–Meier curves for event-free survival regarding the composite endpoint of all-cause death or heart failure (HF) rehospitalization, stratified by pre- to post-TAVI changes in IRVF pattern (4 groups). A significant overall difference was observed among the 4 groups (log-rank *P* = 0.035). Event-free survival was significantly lower in the continuous–discontinuous and discontinuous–discontinuous groups than in the continuous–continuous group (log-rank *P* < 0.05). In Cox proportional hazards analysis, risk was higher in the continuous–discontinuous group (hazard ratio [HR], 3.26; 95% confidence interval [CI], 1.19-8.92; *P* = 0.022) and in the discontinuous–discontinuous group (HR, 2.34; 95% CI, 1.08-5.37; *P* = 0.045), compared with the continuous–continuous group.

Patients were further categorized according to changes in IRVF patterns from pre- to post-TAVI: continuous–continuous (n = 114; 69.5%), discontinuous–continuous (n = 20; 12.2%), continuous–discontinuous (n = 12; 7.3%), and discontinuous–discontinuous (n = 18; 11.0%; Fig. 4). Trends in clinical characteristics and cardiorenal parameters across these 4 groups (Supplemental Table S2) were generally consistent with those observed in the 2-group comparison. Kaplan–Meier analysis showed an overall difference in event-free survival among the 4 groups (Fig. 5; log-rank *P* = 0.035); specifically, both the continuous–discontinuous and discontinuous–discontinuous groups had lower event-free survival than the continuous–continuous group (Fig. 5; each log-rank *P* < 0.05). In the Cox analyses, risk was higher for the continuous–discontinuous (HR, 3.26, 95% CI 1.19-8.92; *P* = 0.022) and discontinuous–discontinuous (HR, 2.34, 95% CI 1.08-5.37; *P* = 0.045) groups, compared with the continuous–continuous group.

### Incremental prognostic value of the post-TAVI IRVF pattern

Adding the post-TAVI IRVF pattern to the baseline model resulted in a small change in the C-index, and likelihood ratio testing, integrated discrimination improvement, and continuous net reclassification improvement were not significant. Decision-curve analysis showed similar net benefit between the models (Supplemental Table S5 and Supplemental Fig. S3).

### Reproducibility of IRVF pattern classification

Inter-observer and intra-observer agreement for IRVF pattern classification were substantial (κ = 0.83 and κ = 1.00, respectively; Supplemental Table S6).

## Discussion

A key finding of this study is that a discontinuous IRVF pattern at discharge was associated with a higher risk of the composite endpoint of all-cause death or HF rehospitalization. In contrast, the pre-TAVI IRVF pattern alone provided limited prognostic discrimination. However, it provided baseline context and enabled assessment of peri-procedural changes in renal venous congestion. Normalization of IRVF after TAVI was associated with more-favourable outcomes, whereas new-onset or persistent discontinuity identified a particularly high-risk subset. An overview of the study is provided in the Central Illustration. These observations suggest that IRVF assessment at discharge may serve as a practical marker of residual congestion for post-TAVI clinical assessment.

**Central Illustration:**
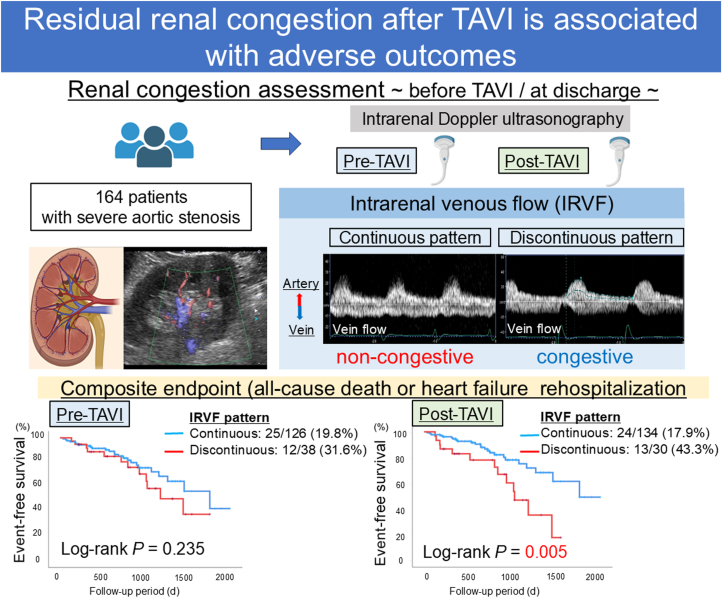
Intrarenal Doppler ultrasonography was performed before transcatheter aortic valve implantation (TAVI) and at discharge in 164 patients with severe aortic stenosis who underwent TAVI. Intrarenal venous flow (IRVF) was assessed as a marker of renal venous congestion. IRVF patterns were categorized as continuous (normal, non-congestive) or discontinuous (renal congestion). A discontinuous IRVF pattern at discharge was associated with a higher risk of the composite endpoint (all-cause death or heart failure rehospitalization) than a continuous pattern (log-rank *P* = 0.005), whereas the pre-TAVI IRVF pattern did not stratify risk.

Prior studies have demonstrated the prognostic utility of IRVF in HF: a discontinuous IRVF pattern reflects right-sided congestion and is associated with adverse outcomes.^9^^,^^10^ In our cohort with severe AS, a post-TAVI discontinuous IRVF pattern was accompanied by higher tricuspid regurgitation pressure gradient and a larger IVC diameter, suggesting residual or new-onset right-sided hemodynamic burden. In HF, IRVF may improve from a discontinuous to a continuous pattern following decongestion in some patients,^16^ whereas persistence of a discontinuous pattern is associated with higher BNP levels, worsening renal function, and adverse outcomes.^17^^,^^18^ Consistent with these observations, some patients in our cohort exhibited post-TAVI improvement in IRVF, yet persistence of a discontinuous pattern at discharge was associated with poorer outcomes.

These findings align with the pathophysiology of severe AS. Elevated left-sided filling pressures can be transmitted retrogradely through the pulmonary circulation, leading to pulmonary hypertension and right ventricular systolic dysfunction. Prognosis worsens as the stage of cardiac damage advances.^19^^,^^20^ After TAVI, early improvement in the cardiac damage stage has been associated with better long-term outcomes, whereas persistence or worsening portends poorer outcomes.^21^^,^^22^ Accordingly, staging after valve unloading captures residual injury in the myocardium, pulmonary vasculature, and right heart and may more directly predict long-term outcomes.^22^ Right-sided load after TAVI is particularly important for prognosis: in the Optimized transCathEter vAlvular iNtervention–Transcatheter Aortic Valve Implantation registry, residual or new-onset pulmonary hypertension after TAVI was associated with higher mortality, compared with normalization.^23^ Right ventricle-pulmonary artery uncoupling after TAVI, reflected by a low ratio of tricuspid annular plane systolic excursion to pulmonary artery systolic pressure (TAPSE/PASP), has been linked to poor prognosis. Recovery of coupling is associated with outcomes comparable to those with normal coupling, whereas persistent or new-onset uncoupling portends the worst survival.^24^ Although TAVI reduces left ventricular pressure overload, right-sided improvement may be limited when left atrial pressure remains elevated due to AF or post-TAVI MR, or when right ventricular dysfunction is advanced. If left atrial pressure stays high, pulmonary hypertension may persist and increase right-sided load. Residual tricuspid regurgitation and volume overload may also worsen systemic venous congestion. As a result, renal venous pressure may remain high, and renal congestion may persist or newly develop after TAVI, especially when patients are not decongested fully before discharge.

To maximize feasibility and reproducibility in our TAVI cohort, we measured IRVF in the segmental renal vein. However, most prior studies evaluated interlobar veins,^9^^,^^10^ and venous flow characteristics may differ by intrarenal sampling site, limiting direct comparability. Future studies should therefore clarify inter-site reproducibility and prognostic performance across sampling sites, and standardize acquisition settings (eg, gain, sweep speed) and interpretation criteria across centres. In addition, prospective longitudinal and interventional studies should test whether optimized decongestive therapy that restores continuous IRVF improves outcomes after TAVI.

Diuretic use at discharge was more frequent in patients with a discontinuous IRVF pattern than in those with a continuous pattern. This likely reflects greater residual congestion and overall disease severity in the discontinuous group, rather than a direct adverse effect of diuretics. Thus, confounding by indication and residual confounding cannot be excluded fully despite adjustment for multiple clinical variables. In our cohort, the median length of hospital stay after TAVI was relatively long, reflecting current practice patterns in Japan.^25^ This prolonged hospitalization may allow more intensive optimization before discharge, including hemodynamic stabilization, careful titration of diuretics and guideline-directed HF medication. In contrast, many Western early-discharge TAVI programs continue some decongestion and medication adjustment after discharge during outpatient follow-up care. Therefore, in short-stay pathways, assessing IRVF during early postdischarge outpatient follow-up evaluation may be a practical alternative, to reflect our findings.

A discontinuous IRVF pattern at discharge is a simple indicator of residual renal congestion after TAVI. Although IRVF assessment at discharge may provide only limited incremental prognostic information beyond standard clinical variables, it can noninvasively evaluate residual renal congestion and may serve as a complementary measure for prognostic assessment after TAVI.

### Study limitations

This single-centre, prospective, observational study has several limitations. First, residual confounding cannot be excluded. In addition, BNP level was dichotomized at the cohort median for subgroup analyses because no established cutoff exists in this setting. Median splits may reduce information and statistical power and may leave residual confounding. Second, IRVF assessment was not feasible in all TAVI patients because of an unstable pre-procedural condition, difficulty maintaining the required position, or early discharge. Therefore, selection bias cannot be excluded fully. Moreover, temporal changes in TAVI practice during the study period may have introduced calendar time bias. Third, we measured IRVF in the segmental renal vein, whereas many prior reports sampled the interlobar vein; thus, comparability may be limited, and the lack of standardized acquisition settings and interpretation criteria may introduce inter-centre variability. In addition, echocardiographic and IRVF assessments were not adjudicated by a centralized core laboratory, and measurement variability cannot be excluded fully. Fourth, measurement timing was not uniform, and the effects of periprocedural therapies and dynamic hemodynamic changes may not have been accounted for fully. In addition, the length of hospital stay in our cohort was relatively long. Therefore, our findings may not be directly generalizable to Western TAVI programs in which early discharge is common, and the external validity should be interpreted with caution. Fifth, the number of patients in the discontinuous group and the overall number of events were relatively small, which may have limited statistical power and precision, particularly for subgroup and interaction analyses. Therefore, these findings should be interpreted cautiously and warrant external validation in larger cohorts. Sixth, comorbidities were defined according to contemporary Japanese guideline criteria. Therefore, the generalizability of our findings to other healthcare settings may be limited and should be assessed with caution. In addition, IRVF assessment using intrarenal Doppler ultrasonography is not routinely feasible in many TAVI centres, which may limit the generalizability of our findings. Finally, IRVF-guided treatment strategies were not tested; therefore, causal inference is limited, and the findings should be considered hypothesis-generating.

## Conclusions

Among patients undergoing TAVI, a discontinuous IRVF pattern at discharge, indicating residual renal congestion**,** was associated with a higher risk of all-cause death or HF rehospitalization. Such risk was comparable to that of patients with a continuous pattern when IRVF normalized, whereas those with new-onset discontinuity (continuous–discontinuous) or persistent discontinuity (discontinuous–discontinuous) were identified as being in a subgroup at particularly high risk.

## Data Availability

The data that support the findings of this study are available from the corresponding author upon reasonable request.

## Footnotes

To access the supplementary material accompanying this article, visit *CJC Open* at https://www.cjcopen.ca/ and at https://doi.org/10.1016/j.cjco.2026.02.016
