# Supplementary material for: Prognostic Impact of Residual Renal Congestion at Discharge After Transcatheter Aortic Valve Implantation
Source: CJC Open. 2026 Feb 27;8(7):831–41. doi: 10.1016/j.cjco.2026.02.016 (PMC13386743; doi:10.1016/j.cjco.2026.02.016)
Supplement: Supplementary Material [file mmc1.docx]

**Supplemental Table S1.** Baseline patient characteristics stratified by pre-TAVI IRVF pattern (*n* = 164)

|  | **Total**  **(*n* = 164)** | **Continuous Group**  **(*n* = 126)** | **Discontinuous Group**  **(*n* = 38)** | ***P*-value** |
| --- | --- | --- | --- | --- |
| **Demographic data** |  |  |  |  |
| Age (years) | 83.4 ± 4.7 | 83.2 ± 4.7 | 84.2 ± 4.8 | 0.279 |
| Female sex (*n*, %) | 106 (64.6) | 75 (59.5) | 31 (81.6) | 0.013 |
| Body mass index (kg/m²) | 22.7 (20.6–25.4) | 22.7 (20.6–25.5) | 22.4 (20.9–25.2) | 0.826 |
| NYHA class III or IV before TAVI (*n*, %) | 37 (22.6) | 21 (16.7) | 16 (42.1) | 0.001 |
| STS score (%) | 4.8 (3.4–6.9) | 4.4 (3.2–6.6) | 6.4 (3.8–8.6) | 0.002 |
| Clinical Frailty Scale ≥ 4 (*n*, %) | 56 (34.1) | 38 (30.2) | 18 (47.4) | 0.050 |
| **Past medical history** |  |  |  |  |
| Hypertension (*n*, %) | 126 (76.8) | 100 (79.4) | 26 (68.4) | 0.161 |
| Diabetes mellitus (*n*, %) | 42 (25.6) | 35 (27.8) | 7 (18.4) | 0.247 |
| Dyslipidemia (*n*, %) | 71 (43.3) | 56 (44.4) | 15 (39.5) | 0.588 |
| Smoking (*n*, %) | 47 (28.7) | 41 (32.5) | 6 (15.8) | 0.045 |
| Peripheral artery disease (*n*, %) | 17 (10.4) | 13 (10.3) | 4 (10.5) | 1.000 |
| Chronic kidney disease (*n*, %) | 110 (67.1) | 80 (63.5) | 30 (78.9) | 0.076 |
| Previous stroke (*n*, %) | 8 (4.9) | 7 (5.6) | 1 (2.6) | 0.683 |
| AF (*n*, %) | 31 (18.9) | 17 (13.5) | 14 (36.8) | 0.001 |
| COPD (*n*, %) | 38 (23.2) | 29 (23.0) | 9 (23.7) | 0.932 |
| OMI (*n*, %) | 7 (4.3) | 3 (2.4) | 4 (10.5) | 0.051 |
| Previous PCI (*n*, %) | 20 (12.2) | 15 (11.9) | 5 (13.2) | 0.784 |
| Previous PMI (*n*, %) | 7 (4.3) | 5 (4.0) | 2 (5.3) | 0.663 |
| **Medication (pre-TAVI)** |  |  |  |  |
| Diuretics (*n*, %) | 66 (40.2) | 46 (36.5) | 20 (52.6) | 0.076 |
| ACE inhibitors or ARBs (*n*, %) | 79 (48.2) | 64 (50.8) | 15 (39.5) | 0.221 |
| Beta-blockers (*n*, %) | 36 (22.0) | 25 (19.8) | 11 (28.9) | 0.235 |
| Calcium channel blockers (*n*, %) | 84 (51.2) | 66 (52.4) | 18 (47.4) | 0.588 |
| **Laboratory data (pre-TAVI)** | |  |  |  |
| BNP (pg/mL) | 224.7 (101.8–498.3) | 223.0 (82.4–470.7) | 369.5 (127.5–551.0) | 0.035 |
| eGFR (mL/min/1.73 m²) | 52.0 (41.0–61.8) | 54.0 (40.5–63.5) | 48.5 (39.8–56.8) | 0.047 |
| Hemoglobin (g/dL) | 11.7 (10.5–12.9) | 11.7 (10.8–13.0) | 10.8 (10.2–12.5) | 0.051 |
| Albumin (g/dL) | 3.9 ± 0.4 | 3.9 ± 0.4 | 3.8 ± 0.5 | 0.408 |
| Sodium (mEq/L) | 138.0 (137.0–140.0) | 138.0 (136.5–140.0) | 139.0 (137.0–140.8) | 0.340 |

| **Echocardiographic data (pre-TAVI)** | | |  | |  | |
| --- | --- | --- | --- | --- | --- | --- |
| LVEF (%) | 62.0 (53.0–67.0) | 63.0 (53.5–67.0) | | 58.5 (48.5–66.8) | | 0.129 |
| E/e′ | 15.0 (11.2–20.2) | 13.6 (10.9–19.6) | | 17.4 (14.8–25.4) | | 0.004 |
| LAD (mm) | 43.5 (40.0–49.0) | 43.0 (39.5–47.0) | | 47.0 (42.0–52.8) | | 0.004 |
| Aortic valve area (cm²) | 0.64 (0.52–0.77) | 0.67 (0.53–0.78) | | 0.59 (0.51–0.69) | | 0.096 |
| AV peak velocity (m/s) | 4.4 (4.1–5.0) | 4.4 (4.1–5.0) | | 4.3 (4.1–4.9) | | 0.257 |
| AV mean pressure gradient (mmHg) | 49.5 ± 14.9 | 49.9 ± 14.1 | | 48.1 ± 17.2 | | 0.558 |
| AR ≥ moderate (*n*, %) | 18 (11.0) | 12 (9.5) | | 6 (15.8) | | 0.372 |
| MR ≥ moderate (*n*, %) | 9 (5.5) | 4 (3.2) | | 5 (13.2) | | 0.032 |
| TRPG (mmHg) | 24.0 (19.8–29.0) | 22.0 (19.0–25.0) | | 27.0 (22.0–33.0) | | 0.023 |
| TAPSE (mm) | 17.9 ± 3.6 | 18.0 ± 3.6 | | 17.8 ± 3.7 | | 0.823 |
| IVC diameter (mm) | 14.0 (12.0–17.0) | 14.0 (12.0–17.0) | | 16.5 (13.0–20.8) | | 0.002 |
| **Procedural characteristics** |  |  | |  | |  |
| Procedural time (min) | 79.0 (64.3–96.8) | 81.0 (64.5–95.5) | | 73.0 (58.8–96.5) | | 0.121 |
| Length of hospital stay (days) | 12.0 (10.0–18.0) | 12.0 (10.0–16.5) | | 16.5 (10.3–20.8) | | 0.007 |
| Contrast (mL) | 67.0 (55.0–83.5) | 70.0 (55.0–81.8) | | 61.0 (55.3–78.3) | | 0.193 |
| Balloon-expandable valve (*n*, %) | 103 (62.8) | 80 (63.5) | | 23 (60.5) | | 0.740 |
| Self-expanding valve (*n*, %) | 61 (37.2) | 46 (36.5) | | 15 (39.5) | | 0.740 |
| **Procedural complications** |  |  | |  | |  |
| Technical success (*n*, %) | 159 (97.0) | 122 (96.8) | | 37 (97.4) | | 1.000 |
| Device success (*n*, %) | 146 (89.0) | 112 (88.9) | | 34 (89.5) | | 1.000 |
| Myocardial infarction (*n*, %) | 1 (0.6) | 1 (0.7) | | 0 (0.0) | | 0.421 |
| Procedural stroke (*n*, %) | 3 (1.8) | 3 (2.4) | | 0 (0.0) | | 1.000 |
| Pacemaker implantation (*n*, %) | 7 (4.3) | 6 (4.8) | | 1 (2.8) | | 1.000 |
| PVL ≥ moderate (*n*, %) | 9 (5.5) | 6 (4.8) | | 3 (7.9) | | 0.434 |

NYHA, New York Heart Association; STS, Society of Thoracic Surgeons; AF, atrial fibrillation; COPD, chronic obstructive pulmonary disease; OMI, old myocardial infarction; PCI, percutaneous coronary intervention; PMI, pacemaker implantation; ACE, angiotensin-converting enzyme; ARB, angiotensin II receptor blocker; BNP, B-type natriuretic peptide; eGFR, estimated glomerular filtration rate; LVEF, left ventricular ejection fraction; E/e′, ratio of early mitral inflow velocity to mitral annular early diastolic velocity; LAD, left atrial diameter; AV, aortic valve; AR, aortic regurgitation; MR, mitral regurgitation; TRPG, tricuspid regurgitation pressure gradient; TAPSE, tricuspid annular plane systolic excursion; IVC, inferior vena cava; PVL, paravalvular leak.

**Supplemental Table S2.** Baseline patient characteristics stratified by pre- to post-TAVI IRVF changes (2 x 2 groups) (*n* = 164)

|  | **Total**  **(*n* = 164)** | **Continuous**–**Continuous Group**  **(*n* = 114)** | **Discontinuous**–**Continuous Group**  **(*n* = 20)** | **Continuous**–**Discontinuous Group**  **(*n* = 12)** | **Discontinuous**–**Discontinuous Group**  **(*n* = 18)** | ***P*-value** |
| --- | --- | --- | --- | --- | --- | --- |
| **Demographic data** | |  |  |  |  |  |
| Age (years) | 83.0  (81.0–87.0) | 83.0  (81.0–86.5) | 83.0  (79.0–85.0) | 83.5  （80.3–86.0） | 87.5  (82.0–88.8) | 0.259 |
| Female sex (*n*, %) | 106 (64.6) | 69 (60.5) | 16 (80.0) | 6 (50.0) | 15 (83.3) | 0.079 |
| Body mass index (kg/m²) | 22.7  (20.6–25.4) | 22.8  (20.3–25.6) | 23.1  (21.6–27.6) | 21.5  （20.4–24.4） | 22.1  (20.3–24.9) | 0.341 |
| NYHA class III or IV (Pre-TAVI) (*n*, %) | 37 (22.6) | 18 (15.8) | 7 (35.0) | 3 (25.0) | 9 (50.0) | 0.006 |
| NYHA class III or IV (Post-TAVI) (*n*, %) | 3 (1.8) | 1 (0.9) | 1 (5.0) | 0 (0.0) | 1 (5.6) | 0.346 |
| STS score (%) | 4.8  (3.4–6.9) | 4.7  (3.2–6.8) | 5.2  (3.7–8.4) | 4.7  （2.9–6.5） | 7.1  (4.7–10.7) | 0.008 |
| Clinical Frailty Scale ≥ 4 (*n*, %) | 56 (34.1) | 33 (28.9) | 7 (35.0) | 5 (41.7) | 11 (61.1) | 0.058 |
| **Medication (pre-TAVI)** | |  |  |  |  |  |
| Diuretics (*n*, %) | 66 (40.2) | 38 (33.3) | 10 (50.0) | 8 (66.7) | 10 (55.6) | 0.040 |
| ACE inhibitors or ARBs (*n*, %) | 79 (48.2) | 60 (52.6) | 8 (40.0) | 4 (33.3) | 7 (38.9) | 0.373 |
| Beta-blockers (*n*, %) | 36 (22.0) | 22 (19.3) | 7 (35.0) | 3 (25.0) | 4 (22.2) | 0.471 |
| Calcium channel blockers (*n*, %) | 84 (51.2) | 63 (55.3) | 13 (65.0) | 3 (25.0) | 5 (27.8) | 0.023 |
| **Medication (post-TAVI)** | |  |  |  |  |  |
| Diuretics (*n*, %) | 68 (41.5) | 39 (34.2) | 10 (50.0) | 9 (75.0) | 10 (55.6) | 0.018 |
| ACE inhibitors or ARB (*n*, %) | 81 (49.4) | 61 (53.5) | 8 (40.0) | 4 (33.3) | 8 (44.4) | 0.408 |
| Beta-blockers (*n*, %) | 44 (26.8) | 29 (25.4) | 4 (20.0) | 6 (50.0) | 5 (27.8) | 0.275 |
| Calcium channel blockers (*n*, %) | 90 (54.9) | 66 (57.9) | 14 (70.0) | 3 (25.0) | 7 (38.9) | 0.038 |
| **Past medical history** | |  |  |  |  |  |
| Hypertension (*n*, %) | 126 (76.8) | 92 (80.7) | 16 (80.0) | 8 (66.7) | 10 (55.6) | 0.096 |
| Diabetes mellitus (*n*, %) | 42 (25.6) | 31 (27.2) | 3 (15.0) | 4 (33.3) | 4 (22.2) | 0.611 |
| Dyslipidemia (*n*, %) | 71 (43.3) | 49 (43.0) | 8 (40.0) | 7 (58.3) | 7 (38.9) | 0.719 |
| Smoking (*n*, %) | 47 (28.7) | 37 (32.5) | 3 (15.0) | 4 (33.3) | 3 (16.7) | 0.259 |
| Peripheral artery disease (*n*, %) | 17 (10.4) | 10 (8.8) | 3 (15.0) | 3 (25.0) | 1 (5.6) | 0.263 |
| Chronic kidney disease (*n*, %) | 110 (67.1) | 72 (63.2) | 15 (75.0) | 8 (66.7) | 15 (83.3) | 0.319 |
| Previous stroke (*n*, %) | 8 (4.9) | 7 (6.2) | 1 (5.0) | 0 (0.0) | 0 (0.0) | 0.583 |
| AF (*n*, %) | 31 (18.9) | 11 (9.6) | 8 (40.0) | 6 (50.0) | 6 (33.3) | < 0.001 |
| COPD (*n*, %) | 38 (23.2) | 27 (23.7) | 4 (20.0) | 2 (16.7) | 5 (27.8) | 0.890 |
| OMI (*n*, %) | 7 (4.3) | 2 (1.8) | 2 (10.0) | 1 (8.3) | 2 (11.1) | 0.116 |
| Previous PCI (*n*, %) | 20 (12.2) | 14 (12.3) | 1 (5.0) | 1 (8.3) | 4 (22.2) | 0.419 |
| Previous PMI (*n*, %) | 7 (4.3) | 4 (3.5) | 2 (10.0) | 1 (8.3) | 0 (0.0) | 0.383 |
| **Laboratory data (pre-/post-TAVI)** | |  |  |  |  |  |
| Pre BNP  (pg/mL) | 224.7  (101.8–498.3) | 180.3  (73.3–371.0) | 473.0  (125.0–536.1) | 454.9  （170.8–842.9） | 369.5  (126.2–568.0) | 0.019 |
| Post BNP (pg/mL) | 115.3  (63.5–243.0) | 82.6  (51.4–190.6) | 187.9  (113.7–279.8) | 283.7  （157.1–536.5） | 114.7  (58.4–253.4) | < 0.001 |
| ΔBNP  (pg/mL) | -80.3  (-278.8–-9.4) | -87.9  (-292.5–-31.8) | -332.3  (-521.6–27.1) | -197.3  （-867.3–-6.6） | -58.7  (-170.4–27.3) | 0.697 |
| Pre eGFR (ml/min/1.73 m²) | 52.0  (41.0–61.8) | 54.0  (41.8–63.3) | 50.0  (42.0–56.8) | 49.0  （39.3–66.3） | 47.0  (33.0–57.5) | 0.232 |
| Post eGFR (ml/min/1.73 m²) | 55.0  (44.5–65.0) | 57.0  (47.0–71.5) | 49.5  (40.8–55.0) | 51.0  （41.0–74.0） | 56.0  (28.0–66.8) | 0.239 |
| ΔeGFR (ml/min/1.73 m²) | 3.0  (-2.0–8.0) | 2.0  (-2.0–6.8) | 3.0  (-2.0–9.0) | 7.0  （-4.0–12.0） | 1.0  (-2.8–4.0) | 0.960 |
| Pre Hemoglobin (g/dL) | 11.7  (10.5–12.9) | 11.7  (10.8–12.7) | 11.4  (10.2–13.7) | 11.3  （10.2–14.2） | 10.3  (10.3–11.7) | 0.149 |
| Post Hemoglobin (g/dL) | 11.0  (10.1–12.0) | 11.0  (10.2–12.1) | 11.3  (9.6–12.1) | 10.5  （9.8–12.0） | 10.4  (9.8–12.0) | 0.388 |
| ΔHemoglobin (g/dL) | -0.8  (-1.6–0.0) | -0.9  (-1.7–-0.2) | -0.2  (-1.5–0.7) | -0.4  （-1.9–-0.4） | -1.0  (-1.9–0.1) | 0.485 |

| Pre Albumin (g/dL) | 3.9  (3.6–4.2) | 4.0  (3.7–4.2) | 4.0  (3.4–4.3) | 3.8  （3.2–4.0） | 3.9  (3.7–4.2) | 0.145 |
| --- | --- | --- | --- | --- | --- | --- |
| Post Albumin (g/dL) | 3.4  (3.1–3.8) | 3.5  (3.1–3.8) | 3.3  (3.0–3.8) | 3.2  （2.9–3.5） | 3.6  (3.2–3.8) | 0.362 |
| ΔAlbumin  (g/dL) | -0.5  (-0.8 – 0.2) | -0.6  (-0.8–-0.3) | -0.5  (-0.9–0.0) | -0.5  （-0.8–-0.1） | -0.5  (-0.7–-0.2) | 0.830 |
| Pre Sodium (mEq/L) | 138.0  (137.0–140.0) | 138.0  (137.0–140.0) | 138.0  (137.0–139.8) | 136.5  （134.0–139.0） | 139.5  (137.0–141.3) | 0.198 |
| Post Sodium (mEq/L) | 138.0  (136.0–140.0) | 137.0  (135.0–138.0) | 137.5  (134.8–140.3) | 137.0  （135.0–138.0） | 138.5  (136.3–139.8) | 0.039 |
| ΔSodium (mEq/L) | -1.0  (-2.0–1.0) | -1.0  (-2.0–1.0) | -1.0  (-2.3–2.0) | 0.0  （-2.0–3.0） | 0.0  (-1.0–2.0) | 0.869 |
| **Echocardiographic data (pre-/post-TAVI)** | | |  |  |  |  |
| Pre LVEF (%) | 62.0  (53.0–67.0) | 63.0  (56.5–67.0) | 58.0  (53.0–67.0) | 63.0  （34.3–67.0） | 59.5  (44.0–67.5) | 0.415 |
| Post LVEF (%) | 61.0  (53.0–65.0) | 61.0  (55.0–65.0) | 51.0  (43.3–62.0) | 63.0  （45.0–70.0） | 62.0  (49.5–64.5) | 0.410 |
| ΔLVEF (%) | 0.0  (-4.0–4.0) | 0.0  (-3.5–3.0) | -3.0  (-7.5–6.3) | 2.0  （-4.0–8.0） | 0.0  (-3.5–4.5) | 0.773 |
| Pre E/e′ | 15.0  (11.2–20.2) | 13.4  (11.0–19.5) | 15.6  (11.7–19.7) | 17.1  （11.8–23.4） | 20.9  (16.5–29.1) | < 0.001 |
| Post E/e′ | 15.9  (11.8–19.7) | 15.1  (11.8–19.2) | 17.3  (11.8–21.7) | 19.7  （13.2–23.4） | 18.2  (16.3–23.8) | 0.027 |
| ΔE/e′ | 0.0  (-3.0–2.0) | 1.0  (-2.0–3.0) | -0.5  (-3.8–2.0) | 0.0  （-4.0–2.0） | 1.0  (-3.0–4.0) | 0.259 |
| Pre LAD (mm) | 43.5  (40.0–49.0) | 42.0  (39.0–46.5) | 47.0  (42.0–55.0) | 48.5  （44.0–52.0） | 46.5  (42.0–48.0) | 0.001 |
| Post LAD (mm) | 44.0  (40.0–49.8) | 43.0  (39.0–47.3) | 45.5  (41.3–55.5) | 47.0  （44.0–53.0） | 47.0  (43.0–51.0) | 0.001 |
| ΔLAD (mm) | 0.0  (-3.0–3.0) | 0.0  (-3.0–3.5) | -2.0  (-3.0–3.3) | 0.0  （-4.0–2.0） | 1.0  (-3.0–4.0) | 0.948 |
| Pre Aortic valve area (cm²) | 0.64  (0.52–0.77) | 0.67  (0.53–0.78) | 0.63  (0.55–0.73) | 0.63  （0.50–0.75） | 0.58  (0.44–0.67) | 0.136 |
| Post Aortic valve area (cm²) | 1.7  (1.5–2.0) | 1.8  (1.5–2.0) | 1.6  (1.5–1.9) | 1.7  （1.5–2.1） | 1.5  (1.4–1.9) | 0.194 |
| Pre AV peak velocity (m/s) | 4.4  (4.1–5.0) | 4.5  (4.2–5.0) | 4.2  (4.0–4.9) | 4.2  (3.6–5.1) | 4.3  (4.1–5.1) | 0.313 |
| Post AV peak velocity (m/s) | 2.0  (1.8–2.3) | 2.0  (1.9–2.3) | 2.0  (1.6–2.2) | 1.8  (1.6–2.3) | 1.9  (1.7–2.3) | 0.231 |
| Pre AV mean pressure gradient (mmHg) | 47.0  (40.0–59.0) | 48.0  (41.0–59.0) | 40.0  (36.0–59.0) | 42.5  （29.3–59.8） | 46.0  (42.3–61.5) | 0.214 |
| Post AV mean pressure gradient (mmHg) | 8.0  (6.0–11.0) | 8.0  (7.0–11.3) | 8.0  (5.3–10.0) | 6.0  （5.0–11.0） | 9.0  (7.0–11.5) | 0.188 |
| Pre AR ≥ moderate (%) | 18 (11.0) | 12 (10.5) | 4 (20.0) | 0 (0.0) | 2 (11.1) | 0.366 |
| Post AR ≥ moderate (%) | 9 (5.5) | 4 (3.5) | 1 (5.0) | 2 (16.7) | 2 (11.1) | 0.182 |
| Pre MR ≥ moderate (%) | 9 (5.5) | 2 (1.8) | 2 (10.0) | 2 (16.7) | 3 (16.7) | 0.011 |
| Post MR ≥ moderate (%) | 6 (3.7) | 1 (0.9) | 1 (5.0) | 2 (16.7) | 2 (11.1) | 0.011 |
| Pre TRPG (mmHg) | 24.0  (19.8–29.0) | 23.0  (19.0–27.0) | 22.0  (18.0–31.0) | 26.5  （20.5–29.0） | 29.0  (25.3–32.5) | < 0.001 |
| Post TRPG (mmHg) | 23.0  (19.0–27.0) | 22.0  (18.0–25.0) | 23.5  (19.3–26.8) | 27.0  （18.0–32.0） | 26.0  (22.0–35.0) | 0.006 |
| ΔTRPG  (mmHg) | -1.0  (-5.0–3.8) | -1.0  (-4.0–3.0) | -0.5  (-6.3–4.3) | 5.0  （-5.0–9.0） | -3.0  (-6.0–1.5) | 0.286 |
| Pre TAPSE  (mm) | 17.7  (15.2–20.4) | 17.9  (15.3–20.5) | 17.5  (15.9–20.2) | 17.8  （14.5–19.3） | 17.2  (14.7–22.0) | 0.865 |
| Post TAPSE (mm) | 18.1  (15.2–21.5) | 18.9  (16.7–22.1) | 16.0  (14.5–18.4) | 15.1  （12.6–19.9） | 17.0  (13.8–22.2) | 0.014 |
| ΔTAPSE (mm) | 0.5  (-2.5–3.3) | 0.8  (-1.6–3.3) | -1.8  (-3.4–3.0) | 0.1  （-3.9–4.1） | -0.4  (-3.1–3.8) | 0.370 |
| Pre IVC (mm) | 14.0  (12.0–17.0) | 14.0  (11.0–16.0) | 18.0  (12.0–19.0) | 16.0  （13.0–20.0） | 17.0  (12.8–21.3) | 0.002 |
| Post IVC (mm) | 14.0  (12.0–17.0) | 14.0  (12.0–16.0) | 13.5  (13.0–16.8) | 19.0  （14.0–21.0） | 15.0  (12.5–20.5) | 0.033 |
| ΔIVC (mm) | 0.0  (-2.0–2.3) | 1.0  (-2.0–3.0) | -2.0  (-4.5–1.0) | 0.0  （-2.0–4.8） | 0.5  (-5.0–1.0) | 0.070 |

| **Procedural characteristics** | |  |  |  |  |  |
| --- | --- | --- | --- | --- | --- | --- |
| Procedural time (min) | 79.0  (64.3–96.8) | 79.0  (64.0–95.0) | 75.0  (68.0–97.0) | 90.5  （71.0–123.0） | 63.0  (53.0–92.8) | 0.140 |
| Length of hospital stay (days) | 12.0  (10.0–18.0) | 11.0  (10.0–15.0) | 19.0  (10.0–23.0) | 16.0  （11.8–24.5） | 13.5  (10.3–18.5) | 0.010 |
| Contrast (mL) | 67.0  (55.0–83.5) | 68.0  (54.5–81.5) | 60.0  (57.0–85.0) | 71.0  （63.0–102.3） | 65.5  (53.0–72.3) | 0.392 |
| Balloon-expandable valve (*n*, %) | 103 (62.8) | 76 (66.7) | 13 (65.0) | 4 (33.3) | 10 (55.6) | 0.131 |
| Self-expanding valve (*n*, %) | 61 (37.2) | 38 (33.3) | 7 (35.0) | 8 (66.7) | 8 (44.4) | 0.131 |
| **Procedural complications** | |  |  |  |  |  |
| Technical success (*n*, %) | 159 (97.0) | 110 (96.5) | 19 (95.0) | 12 (100) | 18 (100) | 0.733 |
| Device success (*n*, %) | 146 (89.0) | 102 (89.5) | 18 (90.0) | 10 (83.3) | 16 (88.9) | 0.932 |
| Procedural stroke (*n*, %) | 3 (1.8) | 3 (2.7) | 0 (0.0) | 0 (0.0) | 0 (0.0) | 0.736 |
| Pacemaker implantation (*n*, %) | 7 (4.3) | 5 (4.4) | 1 (5.0) | 1 (8.3) | 0 (0.0) | 0.751 |
| PVL ≥ moderate (*n*, %) | 9 (5.5) | 4 (3.5) | 1 (5.0) | 2 (16.7) | 2 (11.1) | 0.182 |

Δ, change from pre- to post-TAVI;

BMI, body mass index; NYHA, New York Heart Association; STS, Society of Thoracic Surgeons; AF, atrial fibrillation; COPD, chronic obstructive pulmonary disease; OMI, old myocardial infarction; PCI, percutaneous coronary intervention; PMI, pacemaker implantation; ACE, angiotensin-converting enzyme; ARB, angiotensin II receptor blocker; BNP, B-type natriuretic peptide; eGFR, estimated glomerular filtration rate; LVEF, left ventricular ejection fraction; E/e′, ratio of early mitral inflow velocity to mitral annular early diastolic velocity; LAD, left atrial diameter; AV, aortic valve; AR, aortic regurgitation; MR, mitral regurgitation; TRPG, tricuspid regurgitation pressure gradient; TAPSE, tricuspid annular plane systolic excursion; IVC, inferior vena cava; PVL, paravalvular leak.

**Supplemental Table S3.** Cox proportional hazards and subgroup analyses for the composite outcome of all-cause death or heart failure rehospitalization (37 events/164 patients). Hazard ratios compare the discontinuous vs continuous groups defined by the post-TAVI IRVF pattern at discharge.

|  | Subgroup | *N* | HR | 95% CI | *P***-**value | Interaction  *P***-**value |
| --- | --- | --- | --- | --- | --- | --- |
| Age | ≥ 80.0 | 135 | 2.227 | 1.093–4.540 | 0.027 | 0.301 |
|  | < 80.0 | 29 | 6.292 | 0.394–100.587 | 0.193 |  |
| Sex | Male | 58 | 2.812 | 0.937–8.443 | 0.065 | 0.944 |
|  | Female | 106 | 2.647 | 1.076–6.515 | 0.034 |  |
| BMI | ≥ 22.7 | 83 | 2.749 | 0.553–13.668 | 0.217 | 0.595 |
|  | < 22.7 | 81 | 1.849 | 0.860–3.976 | 0.116 |  |
| Post-TAVI NYHA  Functional class | I or II | 161 | 2.840 | 1.427–5.652 | 0.003 |  |
|  | III or IV | 3 | NA | NA | NA |  |
| STS score | ≥ 4.8 | 86 | 1.709 | 0.729–4.007 | 0.218 | 0.465 |
|  | < 4.8 | 78 | 2.830 | 0.844–9.486 | 0.092 |  |
| Clinical Frailty Scale | ≥ 4.0 | 56 | 1.848 | 0.775–4.408 | 0.166 | 0.610 |
|  | < 4.0 | 108 | 2.637 | 0.808–8.604 | 0.108 |  |
| Hypertension | (+) | 126 | 1.995 | 0.788–5.054 | 0.145 | 0.553 |
|  | (−) | 38 | 3.194 | 1.005–10.150 | 0.049 |  |
| Diabetes mellitus | (+) | 42 | 2.609 | 0.474–14.373 | 0.271 | 0.834 |
|  | (−) | 122 | 2.552 | 1.199–5.428 | 0.015 |  |
| Dyslipidemia | (+) | 71 | 5.063 | 1.458–17.575 | 0.011 | 0.093 |
|  | (−) | 93 | 1.656 | 0.716–3.828 | 0.238 |  |
| Smoking | (+) | 47 | 2.739 | 0.779–10.019 | 0.115 | 0.801 |
|  | (−) | 117 | 2.515 | 1.108–5.709 | 0.027 |  |
| Peripheral artery disease | (+) | 17 | 10.546 | 1.090–102.017 | 0.042 | 0.455 |
|  | (−) | 147 | 2.230 | 1.040–4.778 | 0.039 |  |
| Chronic kidney disease | (+) | 110 | 1.997 | 0.977–4.083 | 0.068 | 0.462 |
|  | (−) | 54 | 4.102 | 0.357–47.062 | 0.257 |  |
| Previous stroke | (+) | 8 | NA | NA | NA |  |
|  | (−) | 156 | 2.644 | 1.319–5.297 | 0.006 |  |
| AF | (+) | 31 | 1.319 | 0.379–4.584 | 0.663 | 0.240 |
|  | (−) | 133 | 3.117 | 1.368–7.100 | 0.007 |  |
| COPD | (+) | 38 | 4.269 | 1.131–16.108 | 0.032 | 0.202 |
|  | (−) | 126 | 1.950 | 0.851–4.469 | 0.114 |  |
| OMI | (+) | 7 | 122.9 | 0.001–21474627.6 | 0.435 | 0.954 |
|  | (−) | 157 | 2.298 | 1.118–4.725 | 0.024 |  |
| Previous PCI | (+) | 20 | 213.587 | 0.003–14193859.8 | 0.344 | 0.931 |
|  | (−) | 144 | 2.230 | 1.059–4.696 | 0.035 |  |
| Previous PMI | (+) | 7 | NA | NA | NA |  |
|  | (−) | 157 | 2.625 | 1.311–5.254 | 0.006 |  |
| Diuretics | (+) | 68 | 1.833 | 0.797–4.218 | 0.154 | 0.871 |
|  | (−) | 96 | 2.582 | 0.696–9.583 | 0.156 |  |
| ACE inhibitors or ARBs | (+) | 81 | 2.317 | 0.711–7.547 | 0.163 | 0.503 |
|  | (−) | 83 | 2.379 | 1.026–5.515 | 0.043 |  |
| Beta-blockers | (+) | 44 | 9.100 | 1.757–47.124 | 0.008 | 0.141 |
|  | (−) | 120 | 1.859 | 0.815–4.238 | 0.140 |  |
| Calcium channel blockers | (+) | 90 | 1.538 | 0.341–6.944 | 0.575 | 0.591 |
|  | (−) | 74 | 2.452 | 1.035–5.808 | 0.042 |  |
| BNP | ≥ 115.3 | 82 | 1.601 | 0.739–3.470 | 0.233 | 0.319 |
|  | < 115.3 | 82 | 3.867 | 0.790–18.927 | 0.095 |  |
| eGFR | ≥ 55 | 88 | 3.788 | 1.260–11.381 | 0.018 | 0.771 |
|  | < 55 | 76 | 1.509 | 0.626–3.637 | 0.059 |  |
| Hemoglobin | ≥ 11.0 | 83 | 3.826 | 1.159–12.627 | 0.028 | 0.316 |
|  | < 11.0 | 81 | 1.798 | 0.772–4.191 | 0.506 |  |
| Albumin | ≥ 3.4 | 96 | 4.348 | 1.260–15.007 | 0.020 | 0.166 |
|  | < 3.4 | 68 | 1.389 | 0.601–3.207 | 0.442 |  |
| Sodium | ≥ 137 | 109 | 3.372 | 1.493–7.618 | 0.003 | 0.273 |
|  | < 137 | 55 | 1.176 | 0.301–4.562 | 0.816 |  |
| LVEF | ≥ 61.0 | 78 | 1.756 | 0.655–4.703 | 0.263 | 0.271 |
|  | < 61.0 | 86 | 3.656 | 1.337–9.997 | 0.012 |  |
| E/e′ | ≥ 15.9 | 84 | 1.646 | 0.679–3.990 | 0.383 | 0.194 |
|  | < 15.9 | 80 | 5.191 | 1.565–17.217 | 0.007 |  |
| LAD | ≥ 44.0 | 86 | 2.779 | 1.188–6.496 | 0.018 | 0.680 |
|  | < 44.0 | 78 | 2.067 | 0.562–7.601 | 0.274 |  |
| Aortic valve area | ≥ 1.72 | 81 | 2.301 | 0.639–8.291 | 0.202 | 0.667 |
|  | < 1.72 | 83 | 2.977 | 1.233–7.187 | 0.015 |  |
| AV peak velocity | ≥ 2.0 | 92 | 2.314 | 0.831–6.442 | 0.108 | 0.870 |
|  | < 2.0 | 72 | 2.334 | 0.896–6.083 | 0.083 |  |
| AV mean pressure gradient | ≥ 8.0 | 99 | 2.431 | 0.941–6.279 | 0.066 | 0.885 |
|  | < 8.0 | 65 | 2.364 | 0.852–6.559 | 0.098 |  |
| MR ≥ moderate | (+) | 6 | NA | NA | NA |  |
|  | (−) | 158 | 2.959 | 1.469–5.960 | 0.002 |  |
| TRPG | ≥ 23.0 | 80 | 2.013 | 0.790–5.129 | 0.143 | 0.399 |
|  | < 23.0 | 71 | 4.164 | 1.273–13.616 | 0.018 |  |
| TAPSE | ≥ 18.1 | 82 | 1.027 | 0.290–3.637 | 0.967 | 0.418 |
|  | < 18.1 | 78 | 3.709 | 1.542–8.921 | 0.003 |  |

| IVC | ≥ 14.0 | 91 | 2.776 | 1.135–6.790 | 0.025 | 0.930 |
| --- | --- | --- | --- | --- | --- | --- |
|  | < 14.0 | 71 | 2.349 | 0.708–7.796 | 0.163 |  |
| Procedural time | ≥ 79.0 | 85 | 3.510 | 1.523–8.091 | 0.003 | 0.431 |
|  | < 79.0 | 79 | 1.713 | 0.494–5.941 | 0.396 |  |
| Length of hospital stay | ≥ 12.0 | 91 | 3.159 | 1.477–6.753 | 0.003 | 0.899 |
|  | < 12.0 | 73 | 0.041 | 0.000–572.603 | 0.512 |  |
| Contrast | ≥ 67.0 | 84 | 2.985 | 1.316–6.774 | 0.009 | 0.520 |
|  | < 67.0 | 80 | 1.676 | 0.422–6.657 | 0.463 |  |
| Valve type | BEV | 103 | 2.705 | 1.037–7.056 | 0.042 | 0.670 |
|  | SEV | 61 | 1.956 | 0.724–5.288 | 0.186 |  |
| Technical success | (+) | 159 | 2.497 | 1.254–4.970 | 0.009 |  |
|  | (−) | 5 | NA | NA | NA |  |
| Device success | (+) | 146 | 2.234 | 1.066–4.679 | 0.033 | 0.103 |
|  | (−) | 18 | 7.071 | 0.634–78.825 | 0.112 |  |
| Myocardial infarction | (+) | 1 | NA | NA | NA |  |
|  | (−) | 163 | 2.569 | 1.297–5.086 | 0.007 |  |
| Procedural stroke | (+) | 3 | NA | NA | NA |  |
|  | (−) | 161 | 2.535 | 1.280–5.019 | 0.008 |  |
| Pacemaker implantation | (+) | 7 | 0.035 | 0.000–49886023 | 0.756 | 0.977 |
|  | (−) | 157 | 2.658 | 1.334–5.296 | 0.005 |  |
| PVL ≥ moderate | (+) | 9 | 1.349 | 0.219–8.316 | 0.747 | 0.688 |
|  | (−) | 155 | 2.418 | 1.136–5.151 | 0.022 |  |

For certain echocardiographic variables, all measurements were attempted; however, some parameters could not be reliably quantified (e.g., suboptimal acoustic windows or inadequate Doppler alignment), resulting in subgroup denominators < 164 (TRPG *n* = 151; TAPSE *n* = 160; IVC diameter *n* = 162).

BMI, body mass index; NYHA, New York Heart Association; STS, Society of Thoracic Surgeons; AF, atrial fibrillation; COPD, chronic obstructive pulmonary disease; OMI, old myocardial infarction; PCI, percutaneous coronary intervention; PMI, pacemaker implantation; ACE, angiotensin-converting enzyme; ARB, angiotensin II receptor blocker; BNP, B-type natriuretic peptide; eGFR, estimated glomerular filtration rate; LVEF, left ventricular ejection fraction; E/e′, ratio of early mitral inflow velocity to mitral annular early diastolic velocity; LAD, left atrial diameter; MR, mitral regurgitation; TRPG, tricuspid regurgitation pressure gradient; TAPSE, tricuspid annular plane systolic excursion; IVC, inferior vena cava; BEV, Balloon-expandable valve; SEV, Self-expanding valve; PVL, paravalvular leak.

**Supplemental Table S4.** Fine–Gray competing-risk regression for HF rehospitalization with all-cause death as a competing event, and cause-specific Cox models for HF rehospitalization and all-cause death.

| **Fine–Gray** |  |  |
| --- | --- | --- |
| HF rehospitalization (death competing event) | HR (95% CI) | *P*-value |
| Discontinuous pattern (vs. continuous pattern), unadjusted | 4.23 (1.51–11.87) | 0.006 |
| Discontinuous pattern (vs. continuous pattern), adjusted for age and sex | 3.23 (1.01–10.35) | 0.019 |
| **Cause-specific Cox models** |  |  |
| HF rehospitalization: 14/164 (8.5%) | HR (95%CI) | *P*-value |
| Discontinuous pattern (vs. continuous pattern), unadjusted | 4.62 (1.60 – 13.30) | 0.005 |
| Discontinuous pattern (vs. continuous pattern), adjusted for age and sex | 4.31 (1.37 – 13.52) | 0.012 |
| All-cause death: 27/164 (16.5%) |  |  |
| Discontinuous pattern (vs. continuous pattern), unadjusted | 1.52 (0.63 – 3.66) | 0.347 |
| Discontinuous pattern (vs. continuous pattern), adjusted for age and sex | 1.07 (0.42 – 2.70) | 0.892 |

**Supplemental Table S5.** Model specification and incremental prognostic performance of adding the post-TAVI IRVF pattern to the baseline clinical model (Model B vs Model A).

A) Model specification

| Model | Covariates |
| --- | --- |
| Model A (baseline clinical model) | age, sex, CFS ≥ 4, AF, logBNP at discharge |
| Model B (extended model) | Model A + post-TAVI IRVF pattern (discontinuous vs. continuous) |

B) Incremental prognostic performance (Model B vs Model A)

|  | Model A | Model B (Model A + post-TAVI IRVF) |
| --- | --- | --- |
| **Discrimination** |  | |
| C-Index | 0.746 | 0.752 (ΔC = +0.006) |
| IDI (95% CI) | 0.005 (-0.010–0.010), *P* = 0.392 | |
| Continuous NRI (95% CI) | 0.120 (-0.312–0.429), *P* = 0.538 | |
| **Calibration** |  | |
| Likelihood ratio test | *P* = 0.414 | |

**Supplemental Table S6.** Inter- and intra-observer agreement for IRVF pattern classification

| **Assessment** | **Cases (N)** | **Agreement, n/N (%)** | **Cohen’s κ** |
| --- | --- | --- | --- |
| Inter-observer agreement | 30 | 28/30 (93.3) | 0.83 |
| Intra-observer agreement | 30 | 30/30 (100) | 1.00 |

Inter-observer and intra-observer reproducibility of IRVF pattern classification (continuous vs. discontinuous) was assessed in a randomly selected subset of 30 examinations. Two experienced sonographers, blinded to clinical data and outcomes, independently reviewed stored Doppler recordings. Agreement was evaluated using Cohen’s κ.

**Supplemental Figure S1.** Love plot of SMDs before and after IPTW


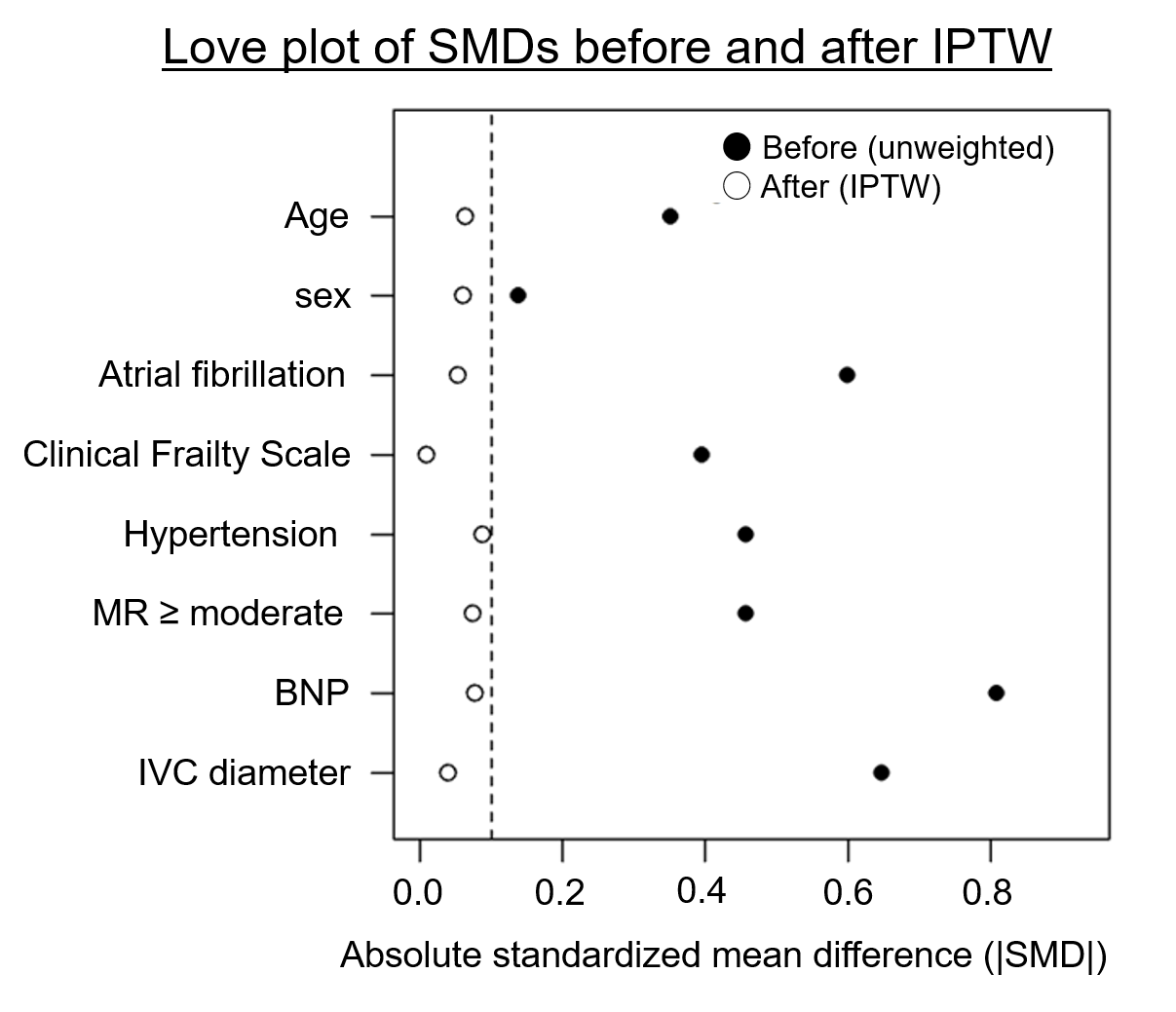


Absolute standardized mean differences (|SMD|) for covariates comparing patients with post-TAVI IRVF patterns (discontinuous vs. continuous) are shown before weighting (unweighted) and after inverse probability of treatment weighting (IPTW). Propensity scores for a discontinuous post-TAVI IRVF pattern were estimated using logistic regression including age, sex, atrial fibrillation, Clinical Frailty Scale (CFS), hypertension, B-type natriuretic peptide (BNP), mitral regurgitation (MR) ≥ moderate, and inferior vena cava (IVC) diameter. After weighting, all absolute SMDs were < 0.10, indicating good covariate balance between groups.

**
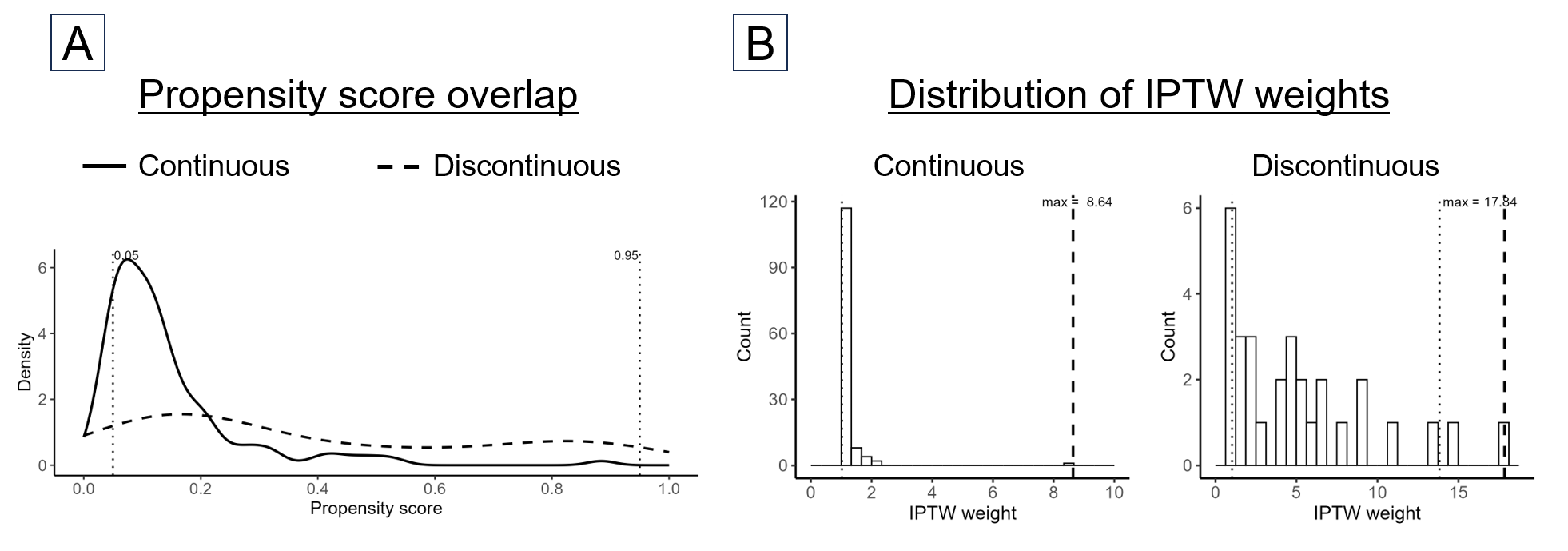
Supplemental Figure S2.** Propensity score overlap and distribution of IPTW weights.

Propensity scores showed overlap between groups, and no patients had extreme propensity scores > 0.95. In the continuous group, 11.4% of patients had propensity scores < 0.05, whereas none had propensity scores < 0.05 in the discontinuous group. IPTW weights were larger in the discontinuous group (maximum 17.8) than in the continuous group (maximum 8.64).

**Supplemental Figure S3**. Decision curve analysis comparing the base model and the base model plus post-TAVI IRVF pattern

**
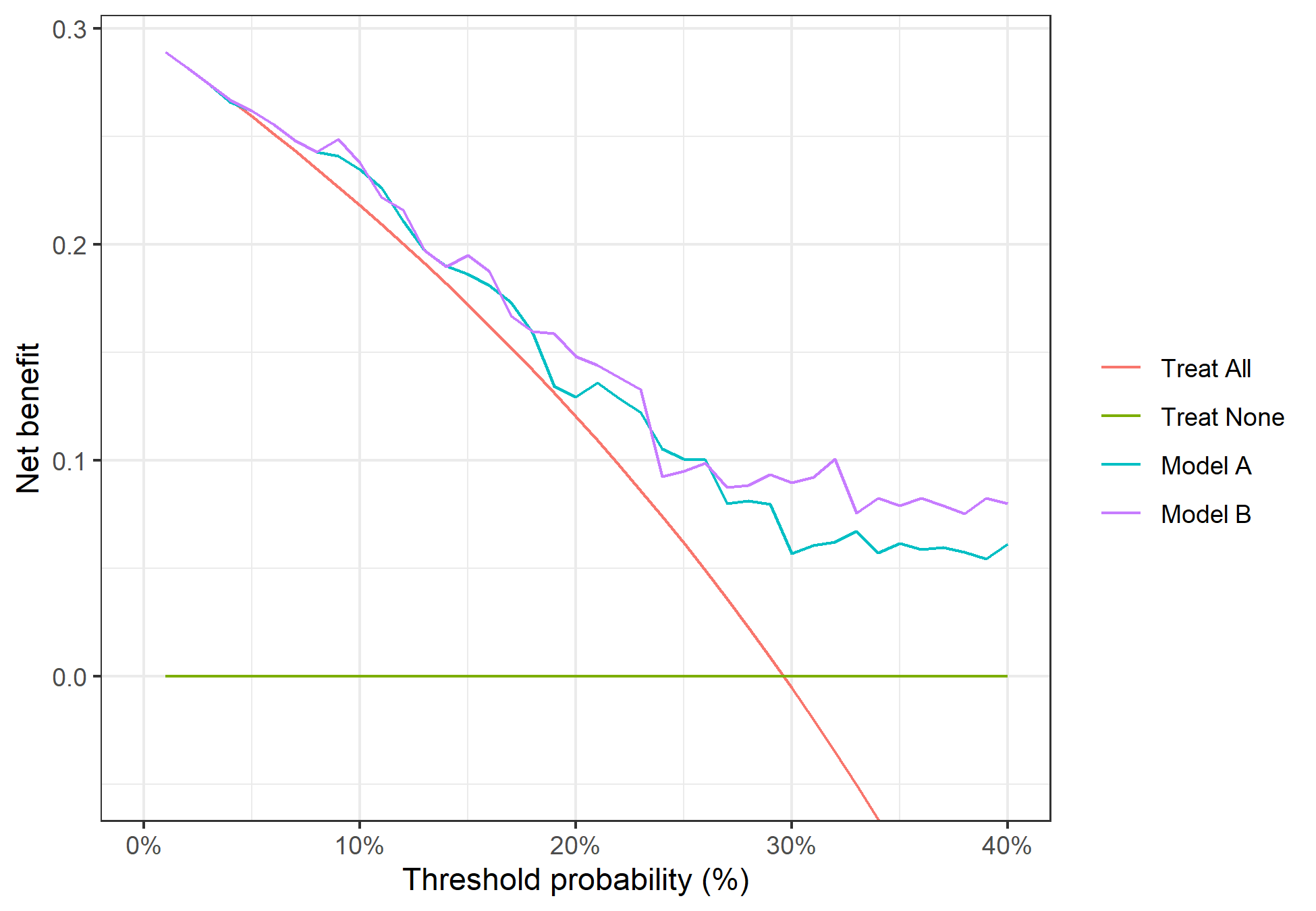
**

Net benefit across threshold probabilities (%) for Model A (baseline clinical model: age, sex, CFS ≥ 4, AF, and log-transformed BNP at discharge) and Model B (extended model: Model A plus the post-TAVI IRVF pattern), compared with treat-all and treat-none strategies. Overall, net benefit was similar between Models A and B.
